# Relaxation Dynamics of Biomass-Derived Copolymers With Promising Gas-Barrier Properties

**DOI:** 10.3389/fchem.2022.921787

**Published:** 2022-06-14

**Authors:** Alejandro Sanz, Amelia Linares, Mari Cruz García-Gutiérrez, Aurora Nogales, Sandra Paszkiewicz, Agata Zubkiewicz, Anna Szymczyk, Tiberio A. Ezquerra

**Affiliations:** ^1^ Instituto de Estructura de La Materia, IEM-CSIC, Madrid, Spain; ^2^ Department of Mechanical Engineering and Mechatronics, West Pomeranian University of Technology, Szczecin, Poland

**Keywords:** polyfuranoates, glass transition, dynamics, random copolymers, dielectric spectroscopy

## Abstract

This article presents an experimental study on the relaxation dynamics of a series of random copolymers based on bio-friendly comonomers with interesting gas barrier properties. We analyze the relaxation response in the glassy and ultraviscous regime of poly (trimethylene furanoate/sebacate) random copolymers via dielectric spectroscopy. We report lower values of dynamic fragility [a dimensionless index introduced in 1985 (Angell, Relaxations in Complex Systems, 1985)] in comparison to popular polyesters widely used in industry, such as poly (ethylene terephthalate), suggesting that the amorphous phase of these furanoate-based polyesters adopt an efficient chain packing. This is consistent with their low permeability to gases. We also discuss on different equations (phenomenological and theory-based approaches) for fitting the temperature-evolution of the alpha relaxation time.

## Introduction

Furanoate-based polyesters are currently the subject of intense research activity, mainly because they are synthesized from biomass derivatives, but also because these materials present attractive gas-barrier, thermal and mechanical properties (Guidotti et al., 2018, 2020). Furan-aromatic polyesters are therefore a realistic alternative to conventional petrochemical polymers (Jiang et al., 2012; Sousa et al., 2015; Tsanaktsis et al., 2015; Genovese et al., 2017). One member of the furan-aromatic polyester family is poly (trimethylene 2,5-furanoate) (PTF), which exhibits a significant reduction in gas transmission as compared to both poly (ethylene terephthalate) (PET) and poly (ethylene 2,5-furanoate) (PEF) (Vannini et al., 2015). Gas transport through polymeric films is mainly governed by the amount of free space not occupied by the chains, and consequently chain-packing efficiency and the transient redistribution of free space by means of localized motions and large-scale segmental rearrangement will play a key role on gas permeability (Shantarovich et al., 2000; Merkel et al., 2002). Regarding the enhanced barrier properties of PTF, a significant reduction in the amplitude of the localized *β* relaxation with respect to PET, has been detected by mechanical spectroscopy measurements (Vannini et al., 2015). The authors rationalized this reduction in terms of hindrance to furan ring-flipping as it was previously proposed by Burgess and co-workers for PEF by means of nuclear magnetic resonance (NMR) spectroscopy (Burgess et al., 2014).

Given that it is well-established that microscopic molecular motions of polymer chains control gas diffusivity, and consequently gas permeability, one way of contributing to understand these fundamental relationships is constructing the so-called relaxation maps. To do so, a powerful tool is broadband dielectric spectroscopy (BDS). A broad range of furanoate-based polyesters has been investigated by BDS, with special focus on disentangling the complexity of the sub-glass dynamics and its connection to gas permeability features (Dimitriadis et al., 2016; Soccio et al., 2017; Genovese et al., 2018; Papamokos et al., 2019; Soccio et al., 2020). Despite in a recent work, Soccio et al. (Soccio et al., 2020) have described the dielectric *β* relaxation of PTF as a bi-modal process, strong arguments to consider this relaxation as mono-modal were previously offered by Genovese et al. (Genovese et al., 2018) and Papamokos et al. (Papamokos et al., 2019). The *β* relaxation of PTF, as a single component, presents an activation energy of approximately 50 *kJ/mol*, value consistent with local motions involving the ester group attached to the furan ring (Soccio et al., 2017). The underlying structural feature related to the mono-modal character of the dynamics in the glassy state of PTF seems to be associated to the stiffness of the furan ring (Burgess et al., 2014; Vannini et al., 2015). There is evidence to conclude that the stiffer the ring, the more tendency for local modes to collapse into a single relaxation (Genovese et al., 2018).

It is also of paramount importance to explore the implications of different microscopic environments for the structural or *α* relaxation in polymeric materials. At isobaric conditions, this universal phenomenon becomes active above the glass transition temperature, *T*_*g*_. The *α* relaxation controls the liquid’s relaxation time and, near *T*_*g*_, is connected to the ultra-viscous flow. Independent of the chemical nature involved, viscous liquids (including polymers) share universal features that precede glass formation, such as the well-known three non’s listed below (Dyre, 2006; McKenna and Simon, 2017; Niss and Hecksher, 2018; Riechers et al., 2022):

• Non-exponential relaxation of equilibrium fluctuations;
• Non-Arrhenius temperature dependence of the *α* relaxation time, *τ*_*α*_;
• Non-linear physical aging (relaxation towards metastable equilibrium).

Regarding the second point, the strong/fragile classification is a mean to quantify on the extent to which glass-forming liquids deviate from the linear Arrhenius law. In other words, the concept of fragility is a measure of the rate at which the characteristic relaxation time (and the corresponding viscosity) changes with temperature. It is a dimensionless index that allows one to classify the liquid’s behavior during vitrification and is defined as follows (Angell, 1985, 1991, 1995; Böhmer et al., 1993):

$$m={\left(\frac{\partial log\tau_{\alpha }}{\partial \left(T_{g}/T\right)}\right)}_{T=T_{g}}$$ (1)

From a theoretical perspective, dynamic fragility *m* has been related to the efficiency of polymer chain packing. In short, the idea is that a disruption of molecular packing at the nanoscale is expected to create more ‘open’ fluids and consequently fragile glass-forming polymers. In contrast, increasing packing efficiency leads to the formation of stronger polymer glasses (Dudowicz et al., 2005b,a). Note that gas permeability strongly depends on the concomitant free volume generated by poorly packed chains. Thus, it is expected the low permeability to small molecules shown, for instance, by polyisobutylene due to its smooth chemical structure and very low value of fragility (*m*∼46) (Mark, 1999; Kunal et al., 2008a). In contrast, much higher permeability to, for instance nitrogen, has been reported for an extremely fragile polyetherimide such as Ultem^®^1,000 (*m*∼214) (Barbari et al., 1988; Simon et al., 1997). For this reason, we consider that a study of the interrelationship between dynamic fragility and gas permeability in furanoate-based polymers deserves to be undertaken.

Here we report on the dielectric relaxation of random copolymers with PTF as the main component, which is copolymerized with poly (trimethylene sebacate) (PTSeb). Our motivation for studying these particular materials deals with the necessity of improving the flexibility of PTF in order to reduce its brittle character, improving in this way its functionality as an eco-friendly packaging polymer. By introducing aliphatic polyester segments into PTF chains we also expect to improve the capabilities of these materials to biodegrade. We consider bio-based PTF-co-PTSeb could represent a viable option to substitute Ecoflex^®^, a well-known BASF product commonly used for the production of mulching foils for agriculture purposes and also used in the field of food packaging. PTF-co-PTSeb films of 100 *μm* thickness and aliphatic co-unit content ranging from 0 to 25 *mol%*, have been examined by BDS with special interest on the sub-glass dynamics and kinetic fragility. We set the discussion of the relaxation response of these random copolymers in the context of their performance as low permeability systems. Lastly, we also discuss on different phenomenological and theoretical interpretations [with and without a dynamic divergence of the Vogel–Fulcher–Tammann form (Vogel, 1921; Fulcher, 1925; Tammann, 1925)] of the temperature dependence of the *α* relaxation time.

## Materials and Methods

### Furanoate-Based Random Copolymers

**FIGURE 1:**
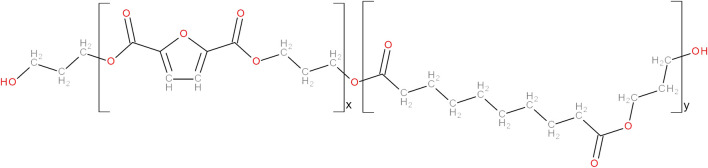
Chemical structure of poly (trimethylene 2,5-furandicarboxylate-co-trimethylene sebacate) (PTF_*x*_-co-PTSeb_*y*_) random copolymer.

Novel fully bio-based PTF-co-PTSeb random copolymers with aliphatic co-unit content in the range 0–25 *mol%* were synthesized by melt polycondensation. Chemical structure is presented in Figure 1, while their composition, molecular weight, polydispersity index and values of *T*_*g*_ are collected in Table 1. A detailed description of the synthesis and chemical structure resolution by conventional spectroscopic techniques has been recently reported (Zubkiewicz et al., 2022).

**TABLE 1:** Chemical composition of PTF-co-PTSeb random copolymers, together with their glass transition temperature as measured by differential scanning calorimetry for compression moulded films. Molecular weight and values of polydispersity are also included.

Sample	Aliphatic Content (mol%)	M_w_ (kg/mol)	Pdi	T_g_ (K)
PTF	0	34.4	2.02	337
PTF95-co-PTSeb5	5	42.8	2.35	323
PTF85-co-PTSeb15	15	43.0	2.44	298
PTF75-co-PTSeb25	25	51.0	2.72	280

### Broadband Dielectric Spectroscopy

Specimens for BDS were melt pressed to ∼ 100 *μm* films, sputtered with gold, mounted between circular gold electrodes (2 cm diameter and 200 mm thickness) and measured using a Novocontrol dielectric system with integrated ALPHA interface. The complex dielectric permittivity, *ɛ*∗(*ω*) = *ɛ*′(*ω*) − *iɛ* ^″^(*ω*), was acquired on heating, after cooling the sample to the lowest temperature measured. Here, *ω* is the angular frequency, and *ɛ*′ and *ɛ* ^″^ are the real and imaginary parts respectively. The temperature is controlled by a nitrogen jet (QUATRO from Novocontrol) with 0.1 K precision during frequency sweeps from 10^−1^–10^6^ Hz.

For dipolar relaxations, the frequency behavior of the complex dielectric permittivity can be described by the empirical Havriliak-Negami equation (Havriliak and Negami, 1967):

$$\varepsilon^{*}\left(\omega ,T\right)=\varepsilon_{\infty }\left(T\right)+\frac{\Delta \varepsilon \left(T\right)}{{\left[1+{\left(i\omega \tau_{HN}\left(T\right)\right)}^{b\left(T\right)}\right]}^{c\left(T\right)}}$$ (2)

In this equation, *ω* is the angular frequency (*ω*=2*πν*), *ɛ*_*∞*_ is the instantaneous or unrelaxed dielectric permittivity, Δ*ɛ* the dielectric strength of the relaxation, *τ*_*HN*_ is a characteristic relaxation time, while *b* and *c* are related to the symmetric and asymmetric broadening of the relaxation peak respectively. As indicated by the notation of Eq. 2, all free parameters are temperature-dependent. We fitted the measured dielectric spectra to a sum of Havriliak-Negami functions by applying a non-linear least square optimization based on the Nelder-Mead simplex method (Lagarias et al., 1998). We used MATLAB routines to perform the whole data analysis.

## Results

### Local and Segmental Dynamics

**FIGURE 2:**
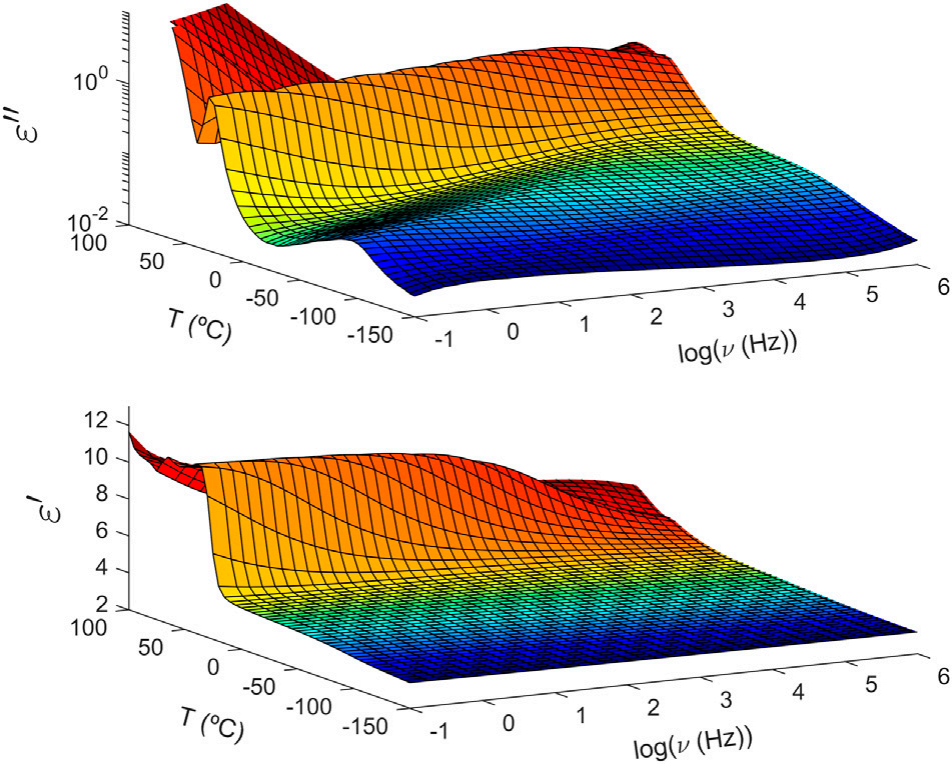
Temperature and frequency (logarithm with base 10) evolution of the complex dielectric permittivity of poly (trimethylene 2,5-furandicarboxylate-co-trimethylene sebacate) (PTF_85_-co-PTSeb_15_) random copolymer. Top: imaginary part. Bottom: real part.

In general terms, the dielectric spectrum of these copolymers follows a similar behaviour, although some differences detected below and above *T*_*g*_ will be discussed below. As illustrated in Figure 2, the imaginary part (top) of the complex dielectric permittivity is dependent upon temperature with three contributions dominating the spectrum. At low temperatures below *T*_*g*_, a low intensity and well-resolved maximum corresponds to the *β* relaxation. Looking at this particular case, PTF_85_-co-PTSeb_15_ shows a bimodal *β* relaxation that can be decomposed into two contributions labeled as *β*_1_ and *β*_2_ in the order of decreasing frequency or increasing temperature. By crossing *T*_*g*_, at intermediate temperatures the *α* relaxation arises as a pronounced peak. At higher temperatures, the intensity of the *α* peak drops significantly, indicating partial crystallization of the sample. Same reduction of intensity is also visible on the step-like decrease of the real part of the permittivity (bottom panel of Figure 2). Finally, at high temperatures and low frequencies, the dielectric loss (*ϵ* ^″^) shows a power-law decrease with frequency due to dc-conductivity induced by the translational mobility of ionic impurities.

**FIGURE 3:**
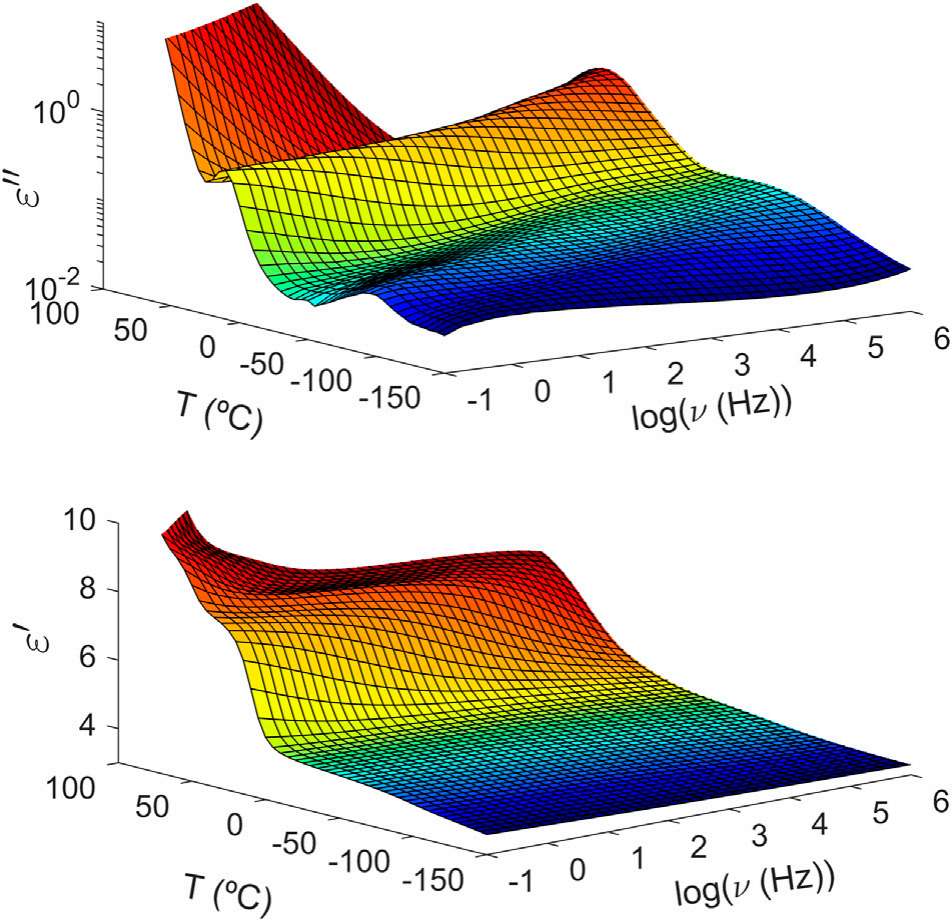
Temperature and frequency (logarithm with base 10) evolution of the complex dielectric permittivity of poly (trimethylene 2,5-furandicarboxylate-co-trimethylene sebacate) (PTF_75_-co-PTSeb_25_) random copolymer. Top: imaginary part. Bottom: real part.

**FIGURE 4:**
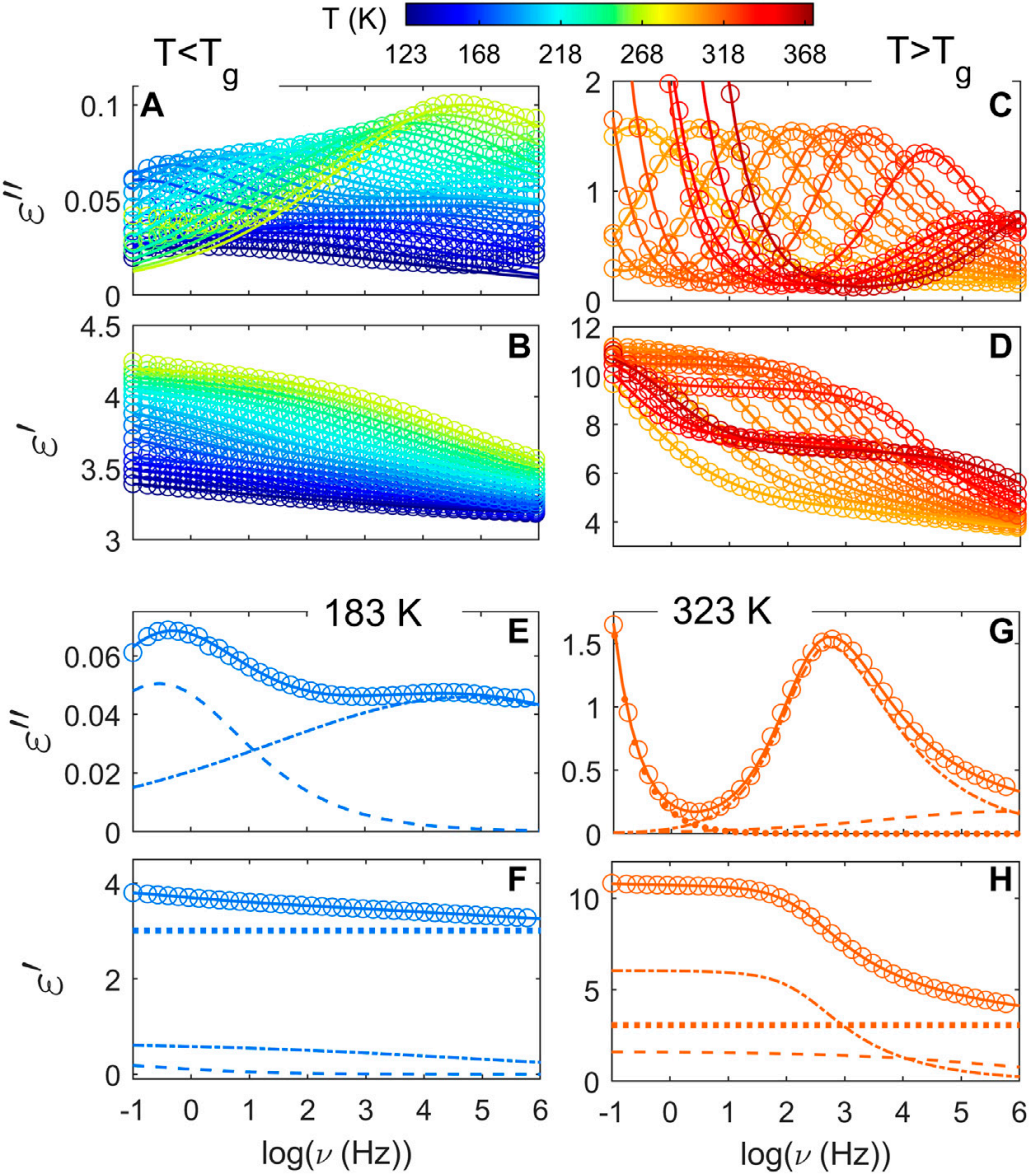
Fits of the complex dielectric permittivity using the Havriliak-Negami formalism for PTF_85_-co-PTSeb_15_. **(A)** and **(B)** panels show selected temperatures below *T*_*g*_. **(C)** and **(D)** panels present the temperature evolution of the *α* relaxation. Below *T*_*g*_, the temperature expands from 123 to 273 K, while above *T*_*g*_ selected temperatures are displayed from 303 to 368 K. Two separate modes contribute to the secondary relaxation as illustrated in panels **(E)** and **(F)** for a temperature of 183 K, with a fast (*β*_1_) and a slow (*β*_2_) relaxation described as dash-dotted and dashed lines respectively. In panels **(G)** and **(H)** the experimental data at 323 K is described as a superposition of three contributions: the prominent *α* relaxation (dash-dotted line), the tail of the *β*_2_ relaxation (dashed line) and a low-frequency contribution of the electrical conductivity (dotted line). Dotted line included in panels **(F)** and **(H)** represents the contribution of *ɛ*_*∞*_. In all cases, solid lines correspond to the total fit of the experimental data to the corresponding model function.

Unlike what is observed in PTF_85_-co-PTSeb_15_, the intensity of the *α* peak does not suffer any drop over the explored temperature range for the copolymer with a 25 mol% of sebacate units. As one may see in Figure 3, the dielectric strength of the alpha relaxation in PTF_75_-co-PTSeb_25_ even seems to increase around *T*_*g*_ + 30 and such result would not follow the proportionality of Δ*ɛ* to reciprocal temperature derived from dielectric relaxation theory (Kremer and Schönhals, 2012). An increase of Δ*ɛ* with temperature is consistent with a progressive mobilization of some fraction of the rigid amorphous phase (RAP) in semicrystalline polymers. The formation and location of RAP, as well as its transformation upon temperature variations have been extensively studied during past decades (Huo and Cebe, 1992; Sanz et al., 2006, Sanz et al., 2010b). Figure 4 shows frequency sweeps at different temperatures for PTF_85_-co-PTSeb_15_ copolymer, where the experimental data are fitted to the Havriliak-Negami function (Eq. 2). Below *T*_*g*_, two symmetric relaxations can fit the spectrum, that is, the values of *c(T)* parameter were fixed to 1 for both the fast (*β*_1_) and slow (*β*_2_) local relaxations over the whole temperature range. Following this phenomenological approach, dielectric data in the glassy state is fitted to the following model function:

$$\varepsilon^{*}\left(\omega ,T\right)=\varepsilon_{\infty }\left(T\right)+\frac{\Delta \varepsilon_{1}\left(T\right)}{1+{\left(i\omega \tau_{\mathrm{HN1}}\left(T\right)\right)}^{b_{1}\left(T\right)}}+\frac{\Delta \varepsilon_{2}\left(T\right)}{1+{\left(i\omega \tau_{\mathrm{HN2}}\left(T\right)\right)}^{b_{2}\left(T\right)}},$$ (3)

where subscripts 1 and 2 stand for local *β*_1_ and *β*_2_ relaxations respectively. By using Eq. 3, we assume that the measured spectrum is a superposition of individual contributions (Schlosser and Schönhals, 1989; Sanz et al., 2004). We report optimum fits as illustrated in panels (a) and (b) of Figure 4. As one would expect, the amplitude of both secondary relaxations increases with temperature. This is a common pattern of localized secondary relaxations which is related to the enhancement in the spatial extent of the constrained motions as temperature increases (Ngai and Paluch, 2004). In panels (e) and (f) we present an example of the fit at 183 K. Separate contribution from *β*_1_ and *β*_2_ processes is shown as dash-dotted and dashed lines respectively. At least for the explored temperature range, we find that *β*_1_ has a broader distribution of relaxation times in comparison to *β*_2_. Values of *b* parameter are nearly constant upon temperature for *β*_1_, ranging from 0.16 to 0.18, while *β*_2_ presents *b* values that decrease with temperature from 0.4 to 0.25 approximately. Similar values for the *b* parameter are obtained for the rest of the members of the PTF-co-PTSeb series. However, it is important to point out that PTF and PTF_95_-co-PTSeb_5_ show a single local relaxation below *T*_*g*_, which corresponds to the *β*_2_ relaxation following the classification scheme introduced before.

The dielectric spectrum changes significantly on crossing *T*_*g*_ as the *α* relaxation enters the experimental frequency window. A full description of the dielectric dispersion in this family of copolymers above *T*_*g*_ requires to account for the *α* and *β*_2_ relaxations, and dc-conductivity. The corresponding function reads as follows:

$$\varepsilon^{*}\left(\omega ,T\right)=\varepsilon_{\infty }\left(T\right)+\frac{\Delta \varepsilon_{2}\left(T\right)}{1+{\left(i\omega \tau_{\mathrm{HN2}}\left(T\right)\right)}^{b_{2}\left(T\right)}}+\frac{\Delta \varepsilon_{\alpha }\left(T\right)}{{\left[1+{\left(i\omega \tau_{HN\alpha }\left(T\right)\right)}^{b_{\alpha }\left(T\right)}\right]}^{c_{\alpha }\left(T\right)}}-i{\left(\frac{\sigma \left(T\right)}{\varepsilon_{0}\omega }\right)}^{s\left(T\right)},$$ (4)

where subscripts 2 and *α* stand for *β*_2_ and *α* relaxations respectively. The last term in Eq. 4 accounts for electrical conduction processes, where *σ*_0_ is the direct current electrical conductivity, *ɛ*_0_ is the vacuum permittivity and *s* is a coefficient depending on the conduction mechanism (0 <s≤ 1). Figure 4 presents examples of simultaneous curve fitting for both the real (panel d) and imaginary (panel c) parts of the complex dielectric function to Eq. 4 at selected temperatures from 303 to 368 K. The fit quality using Eq. 4 is satisfactory over the whole temperature range with the exception of small deviations at high temperatures and low frequencies, mainly for the real part of the permittivity. The presence of ionic charge carriers can provoke interfacial and/or electrode polarization effects that induce this excess of dispersion (Kremer and Schönhals, 2012; Ishai et al., 2013). To compensate for this effect, an additional relaxation was added to the model function at low frequencies as needed. We do not address the physical interpretation of this additional contribution since it goes beyond the scope of the paper.

This general relaxation behavior shows a similar picture independent of the fraction of sebacate units. Nevertheless, some differences between the members of the PTF-co-PTSeb family are listed below:

• PTF and PTF_95_-co-PTSeb_5_ show a single local relaxation, *β*_2_;
• PTF_85_-co-PTSeb_15_ and PTF_75_-co-PTSeb_25_ present two *β* relaxations, *β*_1_ and *β*_2_;
• With the exception of PTF_75_-co-PTSeb_25_, the amplitude of the *α* process shows typical features for a fully amorphous material that undergoes some amount of cold crystallization on heating;
• The temperature-evolution of the dielectric strength for the *α* relaxation in PTF_75_-co-PTSeb_25_ is consistent with a semicrystalline sample.

**FIGURE 5:**
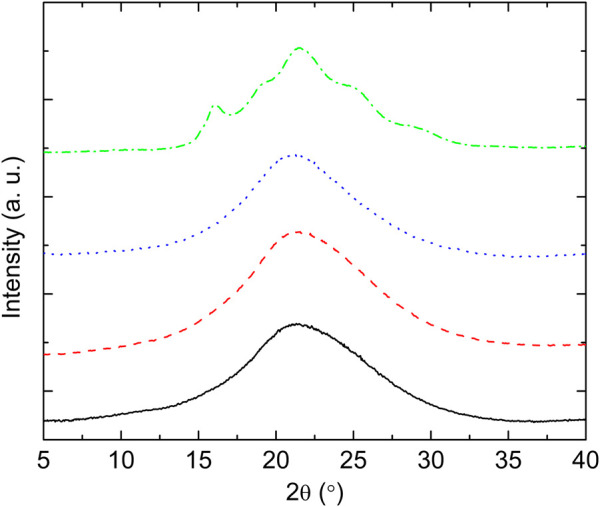
WAXS data of compression molded films of PTF and PTF-co-PTSeb random copolymers. Intensity in arbitrary units is represented as a function of 2*θ*, where *θ* is the scattering angle. Wavelength = 0.154 nm. PTF (—); PTF_95_-co-PTSeb_5_ (- - -); PTF_85_-co-PTSeb_15_ (⋯ ); PTF_75_-co-PTSeb_25_ (–⋅–).

**FIGURE 6:**
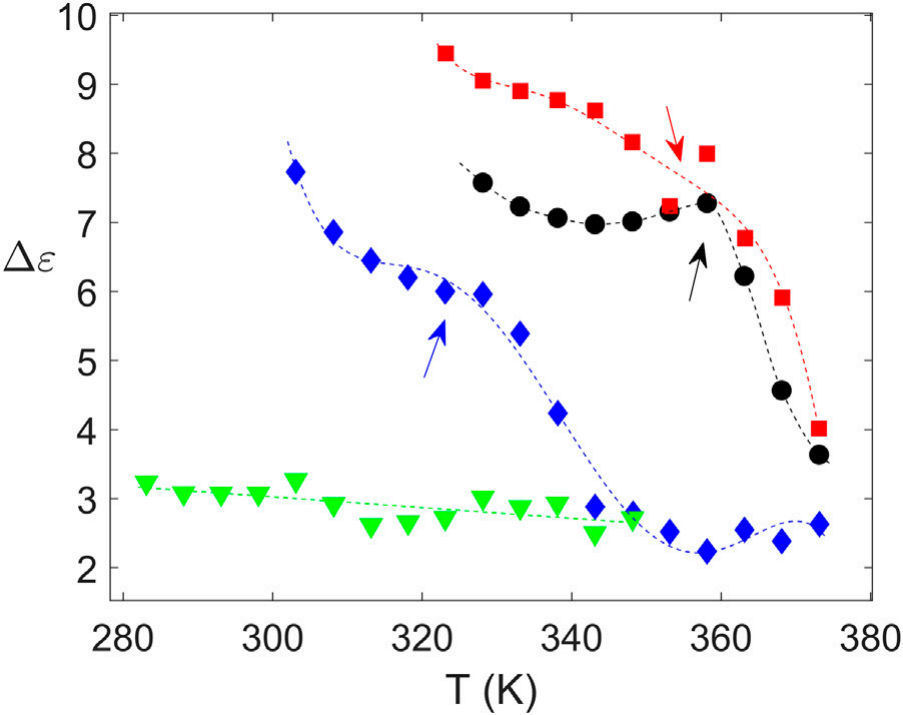
Values of Δ*ɛ* for the *α* relaxation extracted from fittings of the experimental data to Eq. 4. Crystallization onset is highlighted by arrows. Dashed lines are simply guides to eye. PTF (•); PTF_95_-co-PTSeb_5_ (■); PTF_85_-co-PTSeb_15_ (⧫); PTF_75_-co-PTSeb_25_ (▾).

The structural nature of the samples was confirmed by wide angle X-ray scattering (WAXS) as illustrated in Figure 5. Crystalline peaks are observed for PTF_75_-co-PTSeb_25_, while the other samples exhibit a broad halo with absence of crystalline reflections. WAXS data are in complete agreement with the results obtained from dielectric spectroscopy. From fittings to Eq. 4, the dependence with temperature of Δ*ɛ* for the *α* relaxation can be extracted. These data, collected in Figure 6, indicate that PTF_75_-co-PTSeb_25_’s behavior deviates from the other systems, presenting an almost constant dielectric strength with temperature. The lack of any rapid decrease of Δ*ɛ*, together with its lower values at the proximity of *T*_*g*_ in comparison with the other compositions, are strong indicators of the semicrystalline nature of PTF_75_-co-PTSeb_25_. On the contrary, PTF and the copolymers PTF_95_-co-PTSeb_5_ and PTF_85_-co-PTSeb_15_, first show a smooth decrease of Δ*ɛ* as a function of temperature in agreement to the theory of dielectric relaxation (Kremer and Schönhals, 2012). At higher temperatures, one observes an abrupt decrease in the dielectric strength due to cold crystallization. The temperature at which the crystallization begins is very close for PTF and PTF_95_-co-PTSeb_5_, while this onset temperature is around 35 K lower for PTF_85_-co-PTSeb_15_, which is a temperature gap that approximately coincides with the difference between their corresponding values of *T*_*g*_.

From the characteristic time of the Havriliak-Negami function, *τ*_*HN*_, it is possible to calculate the average relaxation time related to the maximum loss frequency by the following relation:

$$\tau =\tau_{HN}{\left[\sin\left(\frac{b\pi }{2+2c}\right)\right]}^{-1/b}{\left[\sin\left(\frac{bc\pi }{2+2c}\right)\right]}^{1/b}$$ (5)

**FIGURE 7:**
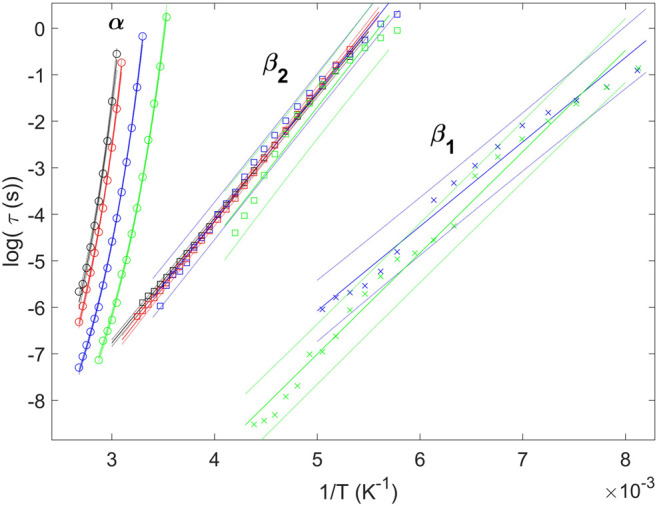
Comparison of the relaxation map between different samples. Labels indicate the location of the observed relaxation processes. VFT and Arrhenius equations are fitted to the *α* and local average relaxation times respectively (solid lines), together with the corresponding 95% confidence intervals (dotted lines). PTF (*α* ◦, *β*_2_ □); PTF_95_-co-PTSeb_5_ (*α* ◦, *β*_2_ □); PTF_85_-co-PTSeb_15_ (*α* ◦, *β*_2_ □, *β*_1_ ×); PTF_75_-co-PTSeb_25_ (*α* ◦, *β*_2_ □, *β*_1_ ×).

We represent the logarithm in base 10 of *τ* with reciprocal temperature for the observed relaxations in Figure 7. The *α* relaxation time follows a super-Arrhenius trend in all cases, as expected for the segmental dynamics of polymers. One observes that, at constant temperatures, the *α* relaxation speeds up with the content of aliphatic units, in full agreement with the reported calorimetric $T_{g}^{\prime }s$ . The most common way to describe the temperature-dependence of the *α* relaxation time is via the Vogel-Fulcher-Tamman (VFT) law. This phenomenological relation reads as follows:

$$\tau =\tau_{0}\exp\left(\frac{DT_{0}}{T-T_{0}}\right),$$ (6)

where *τ*_0_ is a pre-exponential factor with phonon-like time scales, *D* is a strength parameter related to the dynamic fragility and *T*_0_ is the Vogel temperature (divergence at *T* = *T*_0_) (Vogel, 1921; Fulcher, 1925; Tammann, 1925). The VFT equation is a powerful tool for interpolating experimental points and quantifying the steepness of the *α* relaxation plot (Ediger et al., 1996; Sanz and Niss, 2017). For the segmental dynamics (◦), the solid lines in Figure 7 correspond to VFT fits. For all studied samples, the values of *τ*_0_ were fixed to 10^−14^ s (Angell, 1997; Hecksher et al., 2008; Papamokos et al., 2019), while *D* and *T*_0_ were adjustable parameters. On the other hand, a much weaker temperature-dependence is observed for the local relaxations. Values of *τ* for *β*_1_ and *β*_2_ as a function of temperature present a linear trend. These relaxations are normally assigned to localized intramolecular fluctuations. Therefore, continuous lines superimposed to the data points of *β*_1_ and *β*_2_ are fits to the well-known Arrhenius law:

$$\tau =\tau_{\infty }\exp\left(\frac{E_{a}}{RT}\right),$$ (7)

with *E*_*a*_ the activation energy of the relaxation, *R* is the gas constant and *τ*_*∞*_ is a pre-exponential factor that characterizes the high temperature limit of the relaxation time. For the copolymers where the two local relaxations coexist, the linear behavior is less defined for *β*_1_ in comparison to *β*_2_. This leads to broader confidence intervals around the regression line for *β*_1_. Parameters from VFT and Arrhenius fits are listed in Table 2. Regarding the *α* relaxation, the strength parameter *D* presents a slight variation with the sebacate units, while the Vogel temperature *T*_0_ correlates well with the calorimetric *T*_*g*_. Considering the similar values of the activation energy for the *β*_2_ relaxation, including the homopolymer PTF, it seems plausible to assign this relaxation to intramolecular motions of the PTF units. Table 2 shows values of *E*_*a*_ for the *β*_2_ process ranging from 51 to 54 kJ/mol, which are in close agreement with previous works where the activation energy of the single *β* relaxation of PTF has been reported to be 50 ± 3 kJ/mol (Genovese et al., 2018). Given the rigidity of the furan ring, the single *β*_2_ relaxation of PTF involves the rotation of the ester oxygen linked to the aliphatic carbon of the diol subunit, as well as fluctuations around the furan ring carbon and the ester carbon (Genovese et al., 2018). In contrast, one can observe lower activation energies for the *β*_1_ relaxation, indicative of conformational transitions involving more flexible bonds. This suggests that the origin of *β*_1_ is the local relaxation of sebacate units, which would explain why this process is not resolved when the content of PTSeb units is low.

**TABLE 2:** Relaxation parameters and error estimations derived from VFT (*α*) and Arrhenius (*β*_1_ and *β*_2_) fits.

	*α* Relaxation			*β*_1_ Relaxation		*β*_2_ Relaxation	
Sample	D	*T*_0_ (K)	m	*E*_*a*_ (kJ/mol)	Log (*τ*_*∞*_ (s))	*E*_*a*_ (kJ/mol)	Log (*τ*_*∞*_ (s))
PTF	8.3 ± 0.5	258 ± 4	87.2 ± 4.3	—	—	51.3 ± 0.4	−14.8 ± 0.1
PTF95-co-PTSeb5	8.2 ± 0.3	254 ± 2	87.5 ± 2.5	—	—	54.0 ± 0.6	−15.4 ± 0.1
PTF85-co-PTSeb15	8.6 ± 0.2	238 ± 1	84.3 ± 1.5	34.8 ± 3.3	−15.1 ± 1.1	52.8 ± 2.3	−15.2 ± 0.5
PTF75-co-PTSeb25	9.0 ± 0.3	222 ± 2	81.8 ± 2.0	41.7 ± 2.4	−17.9 ± 0.7	54.1 ± 7.7	−15.8 ± 1.9

### Glass Transition Dynamics and Kinetic Fragility

The phenomenological VFT law is the most common way to characterize the slowing down of the *α* relaxation when approaching the glass transition. It is based on the existence of a material dependent temperature *T*_0_ at which the segmental (molecular) relaxation time diverges, albeit the existence of such dynamic divergence has been rigorously challenged (Hecksher et al., 2008). Nevertheless, a good fit to a set of experimental points using the VFT equation (Eq. 6) offers an useful and convenient characterization of the global temperature dependence of the mean relaxation time, specially for the interpolation of data. From the VFT equation, it is also possible to estimate the fragility index (Eq. 1) by using *m* = 16 + (590/*D*), with *D* the strength parameter in Eq. 6 (Böhmer et al., 1993). We here compare the goodness of the VFT fits with two other fitting functions. There are many others, but we make no attempt to provide a full discussion on the alternatives to the VFT law. We simply aim to contribute to raising the polymer community’s awareness about these alternatives.

**FIGURE 8:**
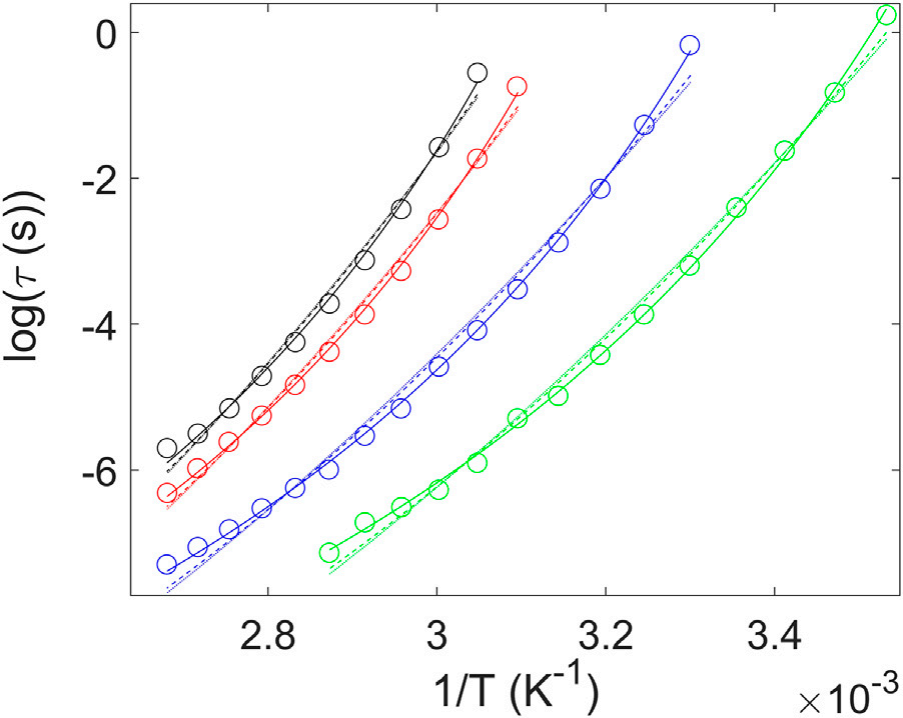
Average relaxation time corresponding to the *α* process as a function of reciprocal temperature for PTF and PTF-co-PTSeb samples. We compare fits of the experimental data using VFT (solid lines), Avramov (dashed lines) and KSZ (dotted lines) functions. In all cases, the pre-exponential factor is held fixed at 10^−14^ s. PTF (◦); PTF_95_-co-PTSeb_5_ (◦); PTF_85_-co-PTSeb_15_ (◦); PTF_75_-co-PTSeb_25_ (◦).

**FIGURE 9:**
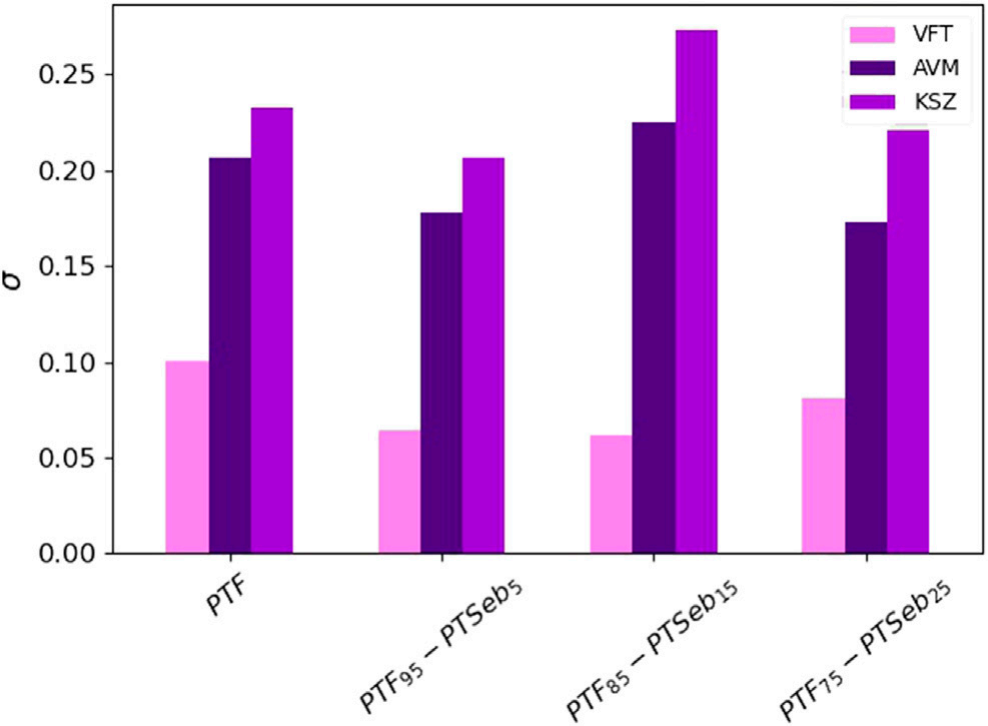
Standard deviation from fits to the temperature-evolution of the *α* relaxation data of the VFT, Avramov and KSZ equations.

To make this comparison, we consider the analytical functions derived from the theoretical model proposed by Avramov-Milchez and KSZ theory (Avramov and Milchev, 1988; Krausser et al., 2015). The Avramov theoretical model describes the structural relaxation time as a single variable function of the total system entropy. This model is based on the argument that structural disorder leads to some probability distribution of activation energies that must be overcome by thermally activated molecular motions. Its functional form reads as follows:

$$\tau =\tau_{0}\exp\left(\frac{B}{T^{n}}\right),$$ (8)

with *τ*_0_ the limiting value at high temperatures, *B* related to the temperature at a reference state and *n* that can be considered a “fragility” parameter defined as *n* = 2*C*_*p*_/*ZR*, where *C*_*p*_ is the heat capacity at constant pressure and *Z* is the degeneracy of the system (available pathways for segmental rearrangement). Inorganic and organic systems have been properly described by Eq. 8 (Avramov, 2000; Hecksher et al., 2008). Krausser, Samwer and Zaccone (KSZ) have proposed a microscopic approach, based on the shoving model (Dyre et al., 1996; Hecksher and Dyre, 2015), where the viscosity is governed by the high frequency shear modulus (Krausser et al., 2015). KSZ function has been found to successfully describe a large variety of glass forming materials with very different chemical nature (Lunkenheimer et al., 2020). The temperature evolution of the structural relaxation time according to the KSZ model is given by:

$$\tau =\tau_{0}\exp\left[\frac{C}{T}\exp\left(-\left(2+\lambda \right)\alpha_{T}T\right)\right],$$ (9)

where *C* is the product of several parameters that cannot be independently estimated by the fits, *τ*_0_ is again the limiting relaxation time at high temperatures, *λ* characterizes the steepness of the repulsive part of the interparticle potential and *α*_*T*_ is the thermal expansion coefficient. The correlation between the dynamic fragility and the product (2 + *λ*)*α*_*T*_ has been experimentally validated for different classes of systems (Lunkenheimer et al., 2020). With these ideas in mind, we have described the temperature dependence of the *α* relaxation time via these three functions (Figure 8). At first sight, the three equations provide an optimum description of the super-Arrhenius behavior of the *α* relaxation. However, in order to analyze in more detail the goodness of the fits, we have estimated the corresponding standard deviation of the residuals *σ*. For this purpose, we have used the expression $\sigma^{2}=1/\left(N-n\right)\sum_{i}{\left(\log\left(\tau_{\mathrm{fit,i}}\right)-\log\left(\tau_{\mathrm{data,i}}\right)\right)}^{2}$ , where *N* is the number of response values and *n* the number of fitted coefficients. A value closer to 0 indicates that the model will fit the data better. Figure 9 shows that the best fits are obtained systematically using the VFT function, while the KSZ model provides the poorest description of the data. This quantitative finding is confirmed by a close visual inspection of Figure 8. In agreement with previous reports for non-polymeric glass formers (Hecksher et al., 2008), our results indicate that describing the data with the VFT law seems to provide better fits, at least considering the frequency range explored here. From now on, we will only consider the relaxation parameters obtained from the VFT fits.

**FIGURE 10:**
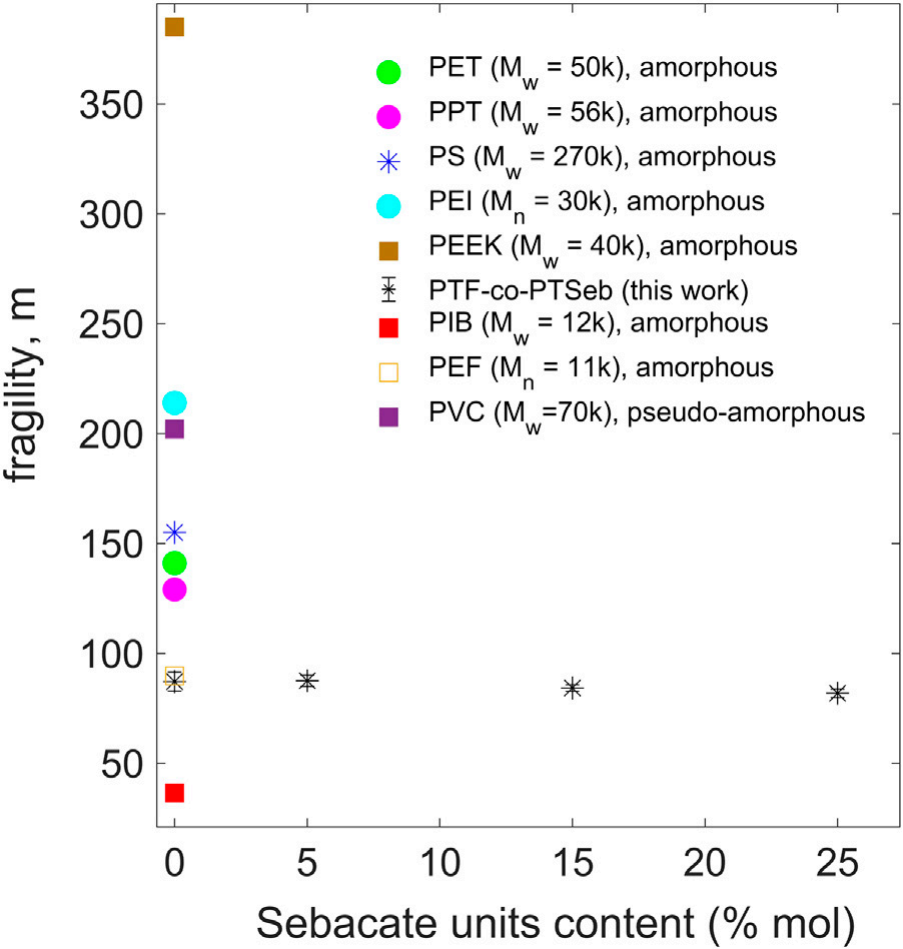
Dynamic fragility *vs*. trimethylene-sebacate content for PTF-co-PTSeb random copolymers. Error bars are almost the same size as the data points. Values of fragility for some representative polymers are included for comparison: Poly (ethylene terephtalate) (PET) (Sanz et al., 2004, 2011), Poly (propylene terephtalate) (PPT) (Sanz et al., 2010a; Soccio et al., 2012), Polystyrene (PS) (Sanz et al., 2015), Polyisobutylene (PIB) (Kunal et al., 2008a), Poly (ether imide) (PEI) (Simon et al., 1997) and Poly (ether ketone) (PEEK) (Nogales et al., 1999). Poly (ethylene 2,5-furanoate) (PEF) (Papamokos et al., 2019). Poly (vinyl chloride) (PVC) (Arbe et al., 2002). Molecular weight is indicated in units of g/mol.

The fragility index *m*, calculated via the strength parameter *D* of the VFT equation, is plotted as a function of the sebacate units fraction in Figure 10. The fragility index presents a slight decrease with the aliphatic content, from 87 ± 4 for PTF to 82 ± 2 for PTF_75_-co-PTSeb_25_, albeit all exhibit values that fall at intermediate fragilities. In polymers containing simple backbones, fragility and *T*_*g*_ are expected to decrease as the amount of flexible units increases (Kunal et al., 2008b), in complete agreement with the findings reported here. However, given the uncertainty estimated for these values and considering the large range of fragilities covered by standard polymers, we can conclude that the fraction of sebacate untis barely modify the fragility index in PTF-co-PTSeb random copolymers, at least for the copolymer composition we study here.

## Discussion

This work presents the first dielectric relaxation study of a novel family of random aliphatic-aromatic copolymers based on PTF and sebacate units. It should be noted that all used co-monomers were derived from fast-renewable resources, such as castor oil and sugars. Additionally, these bio-based materials exhibit good mechanical properties and improved performance as barriers to gases in comparison to the widely used PET (Zubkiewicz et al., 2022). The copolymerization of PTF with the aliphatic sebacate units reduces rigidity and enhances the biodegradability under composting conditions. Given the aforementioned effects, suggesting copolymers of this kind as prospective packaging films is timely, not only because of their promising performance, but also in terms of sustainability.

Our aim is to contribute for a deeper understanding of the microscopic origin of the low permeability to gases in PTF-co-PTSeb random copolymers. It should be kept in mind that chain packing efficiency and localized chain mobility are known to influence on the gas permeability of polymers. Moreover, a generalized entropy theory predicts that a disruption of molecular packing at the nanoscale induces an increase of the dynamic fragility in polymer fluids (Dudowicz et al., 2005b,a). According to this molecular-based theory, the more flexible the macromolecule is, the more efficiency in chain packing and less excess free volume at the glass transition. Using the field-related jargon, the physical properties of fragile materials change abruptly as approaching the glass transition, while strong materials are characterized by a resistance to modification of properties as temperature approaches *T*_*g*_. It is, therefore, well established that polymers with less sterically hindered repeat units can adopt, in general, a more compact chain packing and, consequently, correspond to strong or intermediate glass-formers. In contrast, fragile polymers are commonly characterized by complex and sterically hindered backbones such as PEEK and PEI (see Figure 10) (Colucci and McKenna, 1996; Sokolov et al., 2007). According to these theoretical and phenomenological elements, it is straightforward to rationalize the slight decrease in fragility with increasing the fraction of sebacate units (flexible aliphatic-type chain) in PTF-co-PTSeb random copolymers.

As mentioned before, the resistance to molecular transport across a glassy polymer depends strongly on the free space generated by the inefficient chain packing and the transient voids induced by sub-*T*_*g*_ molecular motions. Poly (ethylene 2,5-furanoate) (PEF) presents better barrier properties in comparison to poly (ethylene terephthalate) (PET). This is a rather striking fact given the larger fractional free volume predicted for PEF (Burgess et al., 2014). The same authors and others explained the lower permeability of PEF in terms of the strong hindrance of furan ring-flipping (Vannini et al., 2015). However, as illustrated in Figure 10, PEF and PTF are stronger materials as compared to PET. That would be consistent with a more compact and efficient chain packing in PEF, PTF and PTF-co-PTSeb copolymers, in comparison to PET. This interpretation is aligned with the study by Papamokos and co-workers that reports a very compact packing of chains for PTF and related poly (n-methylene 2,5- furanoates) due to the stabilization provided by *π* − *π* stacking of the furan rings (Papamokos et al., 2019). The homopolymer PTF and PTF-co-PTSeb random copolymers exhibit superior gas barrier properties to both oxygen and carbon dioxide by comparison with PEF and PET (Zubkiewicz et al., 2022). Since PEF and PTF show similar values of fragility, this seems to indicate that dynamic fragility alone cannot explain the permeability of small molecules through polymeric matrices. This fact is not surprising given that a multitude of factors affect the permeability of polymers (Merkel et al., 2002), including, for example, the thermal history of the sample (Galvani et al., 2007). Nevertheless, dynamic fragility might be considered a good indicator for predicting, at least in a first approximation, the gas barrier properties of polymers. In this respect, several correlations between fragility and other physical properties has been suggested over the past decades (Böhmer et al., 1993; Scopigno et al., 2003; Niss et al., 2007; Sanz et al., 2010a; Lunkenheimer et al., 2020). As in the case reported here, these studies were mostly performed at constant atmospheric pressure, so both temperature and density play a combined role on glass formation. In other words, if the fragility is calculated in this way, it will provide information about the combined effect of temperature and density on the structural relaxation time. Given that both free volume and temperature-activated motions strongly influence on the permeability of polymers, we consider that looking for a relationship between the dynamic fragility and the permeability properties of polymers is meaningful. To ensure this test is sufficiently robust and consistent, one should use some constraints, such as determining the gas transmission rate under comparable conditions and evaluating the fragility index following identical procedures.

## Conclusion

We have described the relaxation behavior of a novel family of random copolyesters based on poly (trimethylene 2,5-furanoate) and poly (trimethylene sebacate). Samples up to a 15% mol fraction of sebacate units were initially amorphous and underwent some cold crystallization on heating. Each subunit is characterized by a single *β* relaxation, with lower activation energy for the trimethylene sebacate component.

The VFT law provides a good description of the temperature evolution of the *α* relaxation time. We report values of dynamic fragility that slightly decrease as the fraction of sebacate units increases. Considering the fragility index of PET as a reference point, the corresponding values for PTF-co-PTSeb random copolymers are significantly lower. These copolymers can be classified as stronger glass-formers using the field-related jargon. Since these copolymers show a lower transmission rate for oxygen and carbon dioxide, it is plausible to claim that they adopt a better and more compact chain packing as compared to PET. The present article underscores the relevance of a tentative correlation between dynamic fragility and the gas transport properties of polymeric matrices that deserves to be tested more carefully in the near future.

## Data Availability

The original contributions presented in the study are included in the article/supplementary material, further inquiries can be directed to the corresponding authors.
